# Reviewer acknowledgement 2012

**DOI:** 10.1186/2045-7022-3-4

**Published:** 2013-02-19

**Authors:** Clive Grattan

**Affiliations:** 1 Norfolk and Norwich University Hospital, Colney Ln, Norwich, NR4 7UY, UK

## Abstract

**Contributing reviewers:**

The editors of *Clinical and Translational Allergy* would like to thank all of our reviewers who have contributed to the journal in Volume 2 (2012).

## 

Werner Aberer

Austria

Ioana Agache

Romania

Ignacio Ansotegui

Spain

Riccardo Asero

Italy

Malin Axelsson

Sweden

Sami Bahna

United States of America

Joan Bartra

Spain

Sevim Bavbek

Turkey

Mehmet Bayram

Turkey

Blanca Bazan-Perkins

Mexico

Carlos Blanco

Spain

Sergio Bonini

Italy

Jean Bousquet

France

Helen A Brough

United Kingdom

Moises A Calderon

United Kingdom

Tomás Chivato

Spain

Cemal Cingi

Turkey

Luis Delgado

Portugal

Pascal Demoly

France

Thomas Eiwegger

Austria

Esben Eller

Denmark

Wytske Fokkens

Netherlands

Gabriele Gadermaier

Austria

Roy Gerth van Wijk

Netherlands

Philippe Gevaert

Belgium

M Hazel Gowland

United Kingdom

Peter Hellings

Belgium

Karin Hoffmann-Sommergruber

Austria

Thomas Holzhauser

Germany

Bogdan Jakiela

Poland

Tuomas Jartti

Finland

Omer Kalayci

Turkey

Allen Kaplan

United States of America

Rebecca Knibb

United Kingdom

George Konstantinou

United States of America

Marcin Kurowski

Poland

Antti Lauerma

Finland

Olga Luengo

Spain

Paraskevi Maggina

Greece

Soili Mäkinen-Kiljunen

Finland

Nadine Marrouche

United Kingdom

Giovanni Melioli

Italy

Joaquim Mullol

Spain

Robert Niven

United Kingdom

Pedro Ojeda

Spain

Oliver Pfaar

Germany

Roland E Poms

Austria

Todor A Popov

Bulgaria

Risto Renkonen

Finland

Claudio Rhyner

Switzerland

Graham Roberts

United Kingdom

Franziska Rueff

Germany

Anna Sala-Cunill

Spain

Peter Schmid-Grendelmeier

Switzerland

Svetlana Sergejeva

Estonia

Masahiro Shoji

Japan

Chrysanthi Skevaki

Greece

Christian Taube

Netherlands

Sanna Toppila-Salmi

Finland

Massimo Triggiani

Italy

Johann C Virchow

Switzerland

Cornelis van Drunen

Netherlands

Carina Venter

United Kingdom

Barbara Yawn

United States of America

